# Research advances in and prospects of ornamental plant genomics

**DOI:** 10.1038/s41438-021-00499-x

**Published:** 2021-04-01

**Authors:** Tangchun Zheng, Ping Li, Lulu Li, Qixiang Zhang

**Affiliations:** Beijing Advanced Innovation Center for Tree Breeding by Molecular Design, Beijing Key Laboratory of Ornamental Plants Germplasm Innovation & Molecular Breeding, National Engineering Research Center for Floriculture, Beijing Laboratory of Urban and Rural Ecological Environment, Engineering Research Center of Landscape Environment of Ministry of Education, Key Laboratory of Genetics and Breeding in Forest Trees and Ornamental Plants of Ministry of Education, School of Landscape Architecture, Beijing Forestry University, Beijing, 100083 China

**Keywords:** Genome, Plant genetics, DNA sequencing

## Abstract

The term ‘ornamental plant’ refers to all plants with ornamental value, which generally have beautiful flowers or special plant architectures. China is rich in ornamental plant resources and known as the “mother of gardens”. Genomics is the science of studying genomes and is useful for carrying out research on genome evolution, genomic variations, gene regulation, and important biological mechanisms based on detailed genome sequence information. Due to the diversity of ornamental plants and high sequencing costs, the progress of genome research on ornamental plants has been slow for a long time. With the emergence of new sequencing technologies and a reduction in costs since the whole-genome sequencing of the first ornamental plant (*Prunus mume*) was completed in 2012, whole-genome sequencing of more than 69 ornamental plants has been completed in <10 years. In this review, whole-genome sequencing and resequencing of ornamental plants will be discussed. We provide analysis with regard to basic data from whole-genome studies of important ornamental plants, the regulation of important ornamental traits, and application prospects.

## Introduction

Genomics is the science of studying genomes. It is used to summarize a branch of genetics involving genome mapping, sequencing, and whole-genome functional analysis. The whole genome is taken as the research object, with a focus on analyzing all of the genetic information in whole genomes of organisms. The main purpose of carrying out genomics research is to interpret the whole-genome sequence, including genomic variations and gene regulation, through mining and expression to gain a deeper understanding of biological mechanisms, formulate more effective breeding strategies, expand the mining breadth and depth of excellent alleles in germplasm resources, and increase the operability for improving complex traits and the efficiency of breeding new varieties.

Ornamental plants, a vital component of agriculture and horticulture, are of great significance for beautifying and improving humans’ living environment, cultivating human sentiment, and promoting structural adjustments in the agricultural industry. The first plant genome to be published was that of *Arabidopsis thaliana* in 2000^1^. With the emergence of next-generation and high-throughput sequencing, sequencing technologies have continuously evolved, while their costs have continuously decreased, facilitating the whole-genome sequencing of many plants. According to incomplete statistics, whole-genome sequencing has been completed for ~400 plants^2^. With this progress, more abundant genetic data are provided for plant diversity studies, enabling breeders to perform comprehensive multidimensional research in the fields of genetics, genomics, and molecular breeding. This brings new development opportunities and driving forces for the breeding of more plants and thus leads to a new revolution of breeding technology. Since genome sequencing of the first ornamental plant (*Prunus mume*) was completed in 2012^3^, whole-genome sequencing of more than 65 ornamental plants has been completed in <10 years. The whole-genome sequencing results from these ornamental plant species have built an enormous resource platform for molecular biology research in ornamental horticulture, which not only contributes to the understanding of genome structure and function in ornamental horticulture but also has substantial guiding significance for exploring the origin and evolution of ornamental plants, mapping and cloning the functional genes of important traits and accelerating the course of molecular breeding.

In this study, the research results from whole-genome sequencing and resequencing of ornamental plants are summarized. We provide a discussion with regard to basic data from whole-genome studies of important ornamental plants, the regulation of important ornamental traits, and application prospects.

## Whole-genome sequences of ornamental plants

**Fig. 1:**
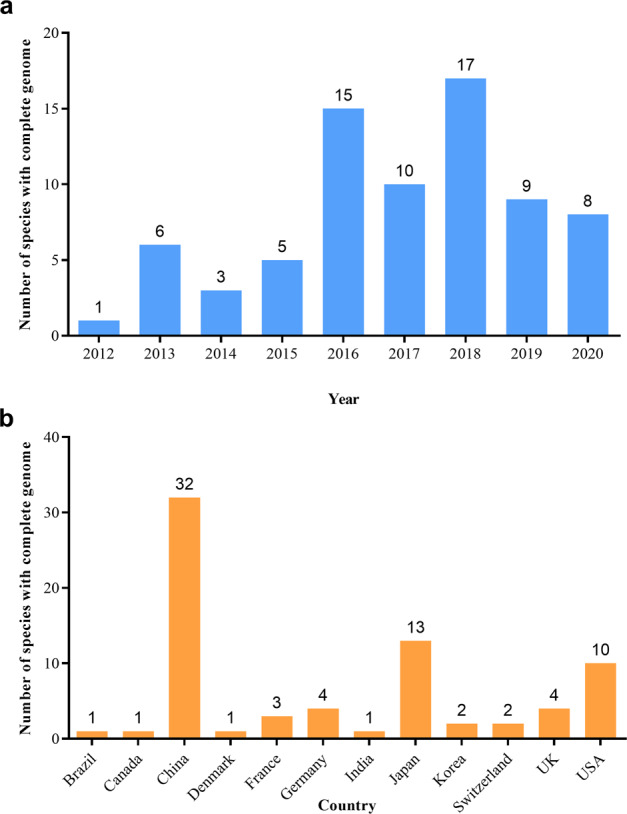
Statistics of ornamental plant species with sequenced genomes. **a** Distribution of genome sequencing for ornamental plants completed from 2012 to 2020; **b** Distribution of genome sequencing for ornamental plants completed in different countries

**Fig. 2:**
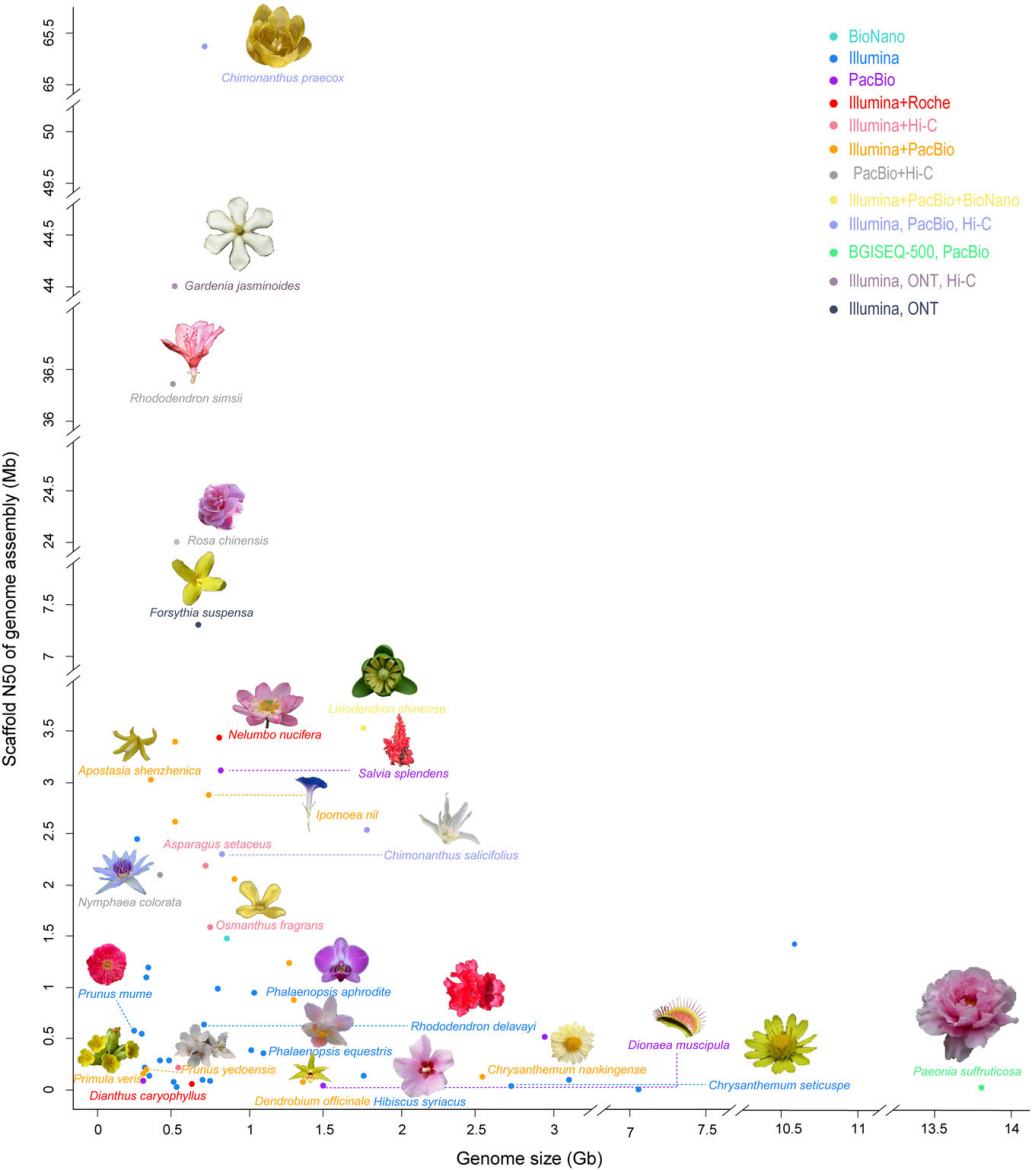
Summary of the representative ornamental plants with complete genome sequencing. The *x*-axis represents the genome size of each plant, while the *y*-axis shows the scaffold N50 of the genome assembly. The sequencing platforms are indicated in different colors

**Fig. 3:**
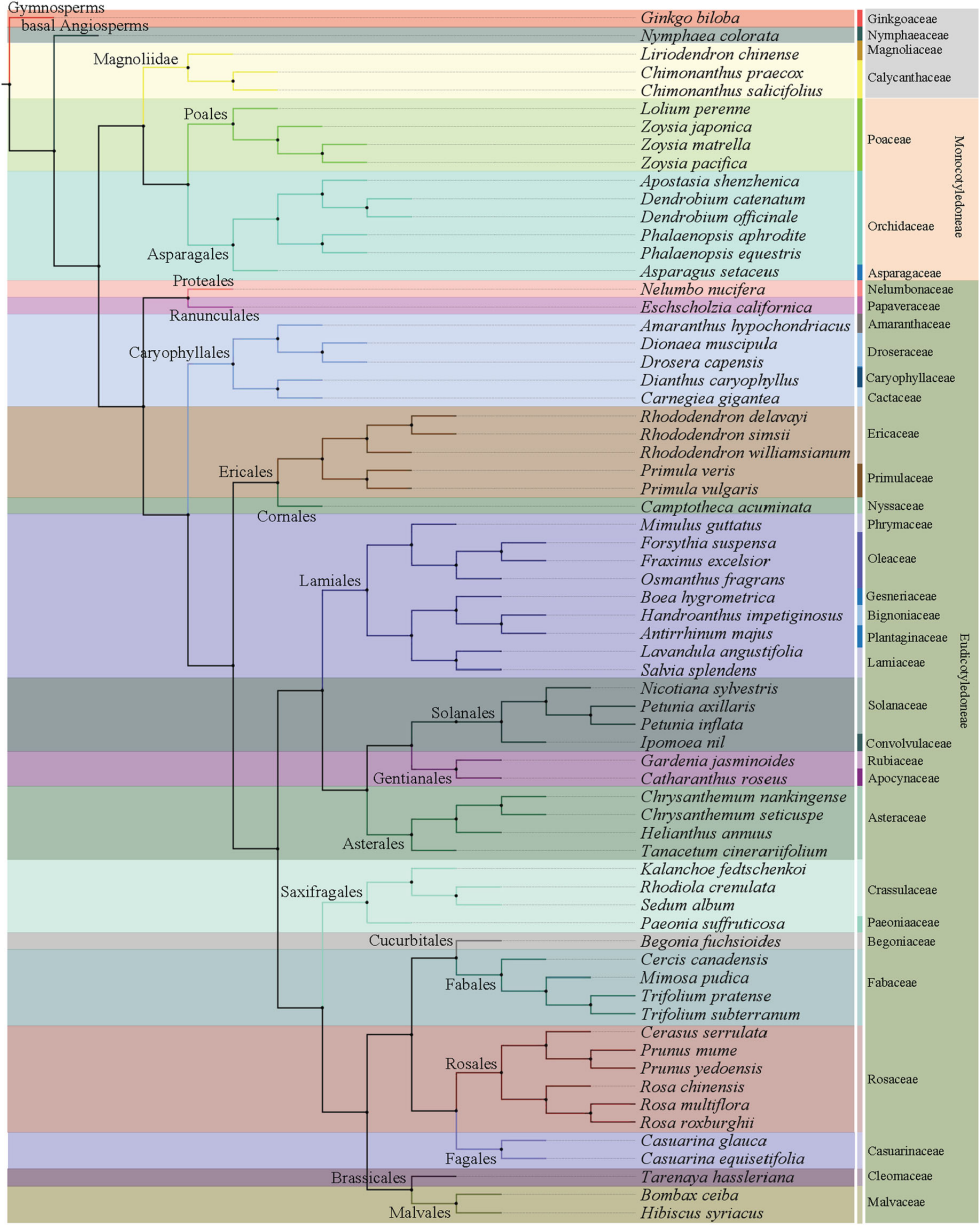
Phylogenetic relationships among ornamental plants with published sequenced genomes. A maximum likelihood (ML) phylogenetic tree was built using low-copy orthologous sequences. All the published ornamental species belong to 21 orders and 35 families. The same background color was used for species in the same family

As of 30 October 2020, the whole-genome sequences and draft genome sequences of 69 ornamental plants have been published, including herbaceous plants, such as carnation (*Dianthus caryophyllus*), phalaenopsis (*Phalaenopsis aphrodite*), orchid (*Apostasia odorata*), sacred lotus (*Nelumbo nucifera*), chrysanthemum (*Dendranthema morifolium*) and *Dionaea muscipula*, and woody plants, such as mei (*Prunus mume*), Yoshino cherry (*Prunus yedoensis*), sweet osmanthus (*Osmanthus fragrans*), peony (*Paeonia suffruticosa*), and Chinese rose (*Rosa chinensis*) (Table 1). The number of sequenced genomes of ornamental plants completed each year significantly increased from 1 in 2012 to 17 in 2018. In particular, more than 10 species were sequenced for three consecutive years from 2016 to 2018 (Fig. 1a). China has independently completed or led genome sequencing for 32 ornamental plants, followed by Japan and the United States, which have also completed the genome sequencing of more than 10 species (Fig. 1b). Considering the sequencing material, except for the double-haploid material with relatively high homozygosity used for *R. chinensis*^4,5^, wild diploids or cultivars with relatively unclear genetic backgrounds and low heterozygosity were used for all of the other plants. Long-read sequencers in combination with optical maps^6^ are used to generate high-quality chromosome-level genome assemblies. For ornamental plants, the PacBio RS II system was first applied for the construction of the 1.27 Gb genome assembly of *Dendrobium officinale*^7^. Long-range scaffolding techniques such as high-throughput chromosome conformation capture (Hi-C) facilitate chromosome-scale assembly of contigs. In this respect, recently built genome assemblies of *Rosa chinensis* (515 Mb) have a contig N50 of 24 Mb, which is one of the most comprehensive plant genomes^4^. In consideration of the comprehensive utilization of Illumina HiSeq, Nanopore, PacBio, and Hi-C technologies, the contig N50 values of *Gardenia jasminoides* and *Chimonanthus praecox* can reach 44 and 65.35 Mb, respectively, which was unthinkable five years ago^8,9^. Generally, the sequencing technology that is predominantly used is next-generation sequencing on the Illumina platform (HiSeq 2000/2500/4000 and HiSeq X ten), coupled with third-generation sequencing (PacBio and Nanopore) and Hi-C technology. The assembled genome size of sequenced ornamental plants ranges from 237 Mb to 13.79 Gb with a scaffold N50 ranging from 13.8 Kb to 65.35 Mb (Fig. 2). We constructed phylogenetic trees for all species with a published genome, which belong to 21 orders and 35 families (Fig. 3). The representative species in Rosaceae, Orchidaceae, and Asteraceae for which high-quality sequencing has been completed were described and discussed.

**Table 1:** List of current genome sequencing progress in ornamental plants

Code	Date	Species	Estimated genome size	Chromosome number	Assembled genome size	Number of scaffolds	Scaffold N50	Number of predicted genes	Sequencing platform	Objects/goals	Country of main contributor	Reference
1	27-Dec-12	*Prunus mume*	280 Mb	2*n* = 16	237 Mb	29,989	577.8 Kb	31,390	Illumina GA II	Early blooming, endodormancy, bacterial infection, biosynthesis of flower scent.	China	^3^
2	10-May-13	*Nelumbo nucifera*	929 Mb	2*n* = 16	804 Mb	3,605	3.435 Mb	26,685	Illumina HiSeq 2000, Roche 454	Aquatic lifestyle	China, USA	^21^
3	17-Jun-13	*Nicotiana sylvestris*	2.64 Gb	2*n* = 24	2.22 Gb	253,984	79.7 Kb	38,940	Illumina HiSeq 2000	Terpenoid metabolism, alkaloid metabolism, and heavy metal transport	Switzerland	^22^
4	27-Aug-13	*Tarenaya hassleriana*	300 Mb	2*n* = 20	290 Mb	98	551.9 Kb	28,917	Illumina HiSeq 2000	Floral developmental, self-incompatibility	China, Netherlands	^23^
5	11-Oct-13	*Nelumbo nucifera*	879 Mb	2*n* = 16	792 Mb	3,031	986.5 Kb	36,385	Illumina HiSeq 2000	Seed formation, embryonic development, seed dormancy, starch synthesis	China	^24^
6	26-Nov-13	*Mimulus guttatus*	–	2*n* = 28	321.7 Mb	2,216	1.1 Mb	26,718	Illumina HiSeq 2000	Recombination activity	USA	^25^
7	17-Dec-13	*Dianthus caryophyllus*	670 Mb	2*n* = 30	622 Mb	45,088	60.74 Kb	43,266	Illumina HiSeq 1000, GS FLXþ	Phenylpropanoid biosynthetic, betalain/chlorophyll and carotenoid synthesis, disease resistance, ethylene/carbohydrate metabolism, and cell wall modification during flower opening, floral scent	Japan	^26^
8	28-Jul-14	*Amaranthus hypochondriacus*	466 Mb	2*n* = 32	465.2 Mb	4,897	35.09 Kb	24,829	Illumina HiSeq 2000	Lysine biosynthetic pathway	India	^27^
9	24-Nov-14	*Phalaenopsis equestris*	1.16 Gb	2*n* = 38	1.09 Gb	523	359.12 Kb	29,431	Illumina HiSeq 2000	Crassulacean acid metabolism, MADS-box genes	China, Belgium	^13^
10	23-Dec-14	*Dendrobium officinale*	1.27 Gb	2*n* = 38	1.35 Gb	33,364	76.49 Kb	35,567	Illumina HiSeq 2000, PacBio RS II	MADS-box genes, morphology of the flower, polysaccharides, alkaloids	China	^7^
11	24-Jan-15	*Primula veris*	479.22 Mb	2*n* = 22	301.8 Mb	9,002	163.95 Kb	19,507	Illumina HiSeq 2000, PacBio RS II	Floral morphs	Switzerland, Norway	^28^
12	11-Mar-15	*Catharanthus roseus*	–	2*n* = 16	523 Mb	79,302	26.25 Kb	33,829	Illumina HiSeq 2000	Monoterpene indole alkaloid pathway	UK, USA	^29^
13	5-May-15	*Boea hygrometrica*	1.69 Gb	–	1.55 Gb	520,969	110.99 Kb	49,374	Illumina HiSeq 2000, Roche 454	Desiccation tolerance	China	^30^
14	26-Sep-15	*Lolium perenne*	1.99 Gb	2*n* = 14	1.13 Gb	48,415	70.1 Kb	28,455	Illumina HiSeq 2000	Pollen allergens, self-incompatibility mechanism	Denmark	^31^
15	30-Nov-15	*Trifolium pratense*	420 Mb	2*n* = 14	309 Mb	39,904	223 Kb	40,868	Illumina HiSeq 2000	Forage nutrition traits	UK	^32^
16	12-Jan-16	*Dendrobium catenatum*	1.11 Gb	2*n* = 38	1.01 Gb	723	391.46 Kb	28,910	Illumina HiSeq 2000	Polysaccharide synthase, MADS-box genes	China, Belgium	^16^
17	5-Feb-16	*Rosa roxburghii*	480.97 Mb	2*n* = 14	409.36 Mb	627,554	1.48 Kb	22,721	Illumina HiSeq 2500	Ascorbate metabolism	China	^33^
18	14-Mar-16	*Zoysia japonica*	340 Mb	2*n* = 40	334.38 Mb	11,786	2.37 Mb	59,271	Illumina HiSeq 2000, MiSeq	Comparative genome	Japan	^34^
19	14-Mar-16	*Zoysia matrella*	423 Mb	2*n* = 40	563.44 Mb	13,609	108.90 Kb	95,079	Illumina HiSeq 2000, MiSeq	Comparative genome	Japan	^34^
20	14-Mar-16	*Zoysia pacifica*	302 Mb	2*n* = 40	397.01 Mb	11,428	111.45 Kb	65,252	Illumina HiSeq 2000, MiSeq	Comparative genome	Japan	^34^
21	12-May-16	*Phalaenopsis orchid*	3.45 Gb	2*n* = 38	3.1 Gb	149,151	100.94 Kb	41,153	Illumina HiSeq 2000	Labellum organ development, flowering-time genes	China, Australia	^14^
22	27-May-16	*Petunia axillaris*	1.4 Gb	2*n* = 14	1.26 Gb	83,639	1.24 Mb	32,928	Illumina HiSeq 2000, PacBio RS II	Floral color, pollination	USA, Switzerland	^35^
23	27-May-16	*Petunia inflata*	1.4 Gb	2*n* = 14	1.29 Gb	136,283	884.43 Kb	36,697	Illumina HiSeq 2000, PacBio RS II	Floral color, pollination	USA, Switzerland	^35^
24	13-Jul-16	*Drosera capensis*	293 Mb	–	264 Mb	13,142	82.65 Kb	–	Illumina HiSeq 2500	3D structures of cysteine protease	USA	^36^
25	28-Jul-16	*Amaranthus hypochondriacus*	466 Mb	2*n* = 32	377 Mb	3,518	371 Kb	23,059	Illumina HiSeq 2500	Systematic evolution	USA	^37^
26	22-Aug-16	*Trifolium subterranum*	552.4 Mb	2*n* = 16	471.8 Mb	27,424	287.6 Kb	42,706	Illumina HiSeq 2000	Evolutionary divergence	Japan	^38^
27	8-Nov-16	*Ipomoea nil*	750 Mb	2*n* = 30	734.8 Mb	3,416	2.88 Mb	42,783	Illumina HiSeq2500, PacBio RS II	Dwarf trait	Japan	^39^
28	21-Nov-16	*Ginkgo biloba*	10 Gb	2*n* = 24	10.61 Gb	6,459,773	1.36 Mb	41,840	Illumina HiSeq 2000/4000	Multiple defense mechanisms, resistant genes	China	^40^
29	21-Dec-16	*Hibiscus syriacus*	1.9 Gb	2*n* = 80	1.75 Gb	77,492	140 Kb	87,603	Illumina HiSeq 2000	Flowering time, disease resistance	Korea	^41^
30	26-Dec-16	*Fraxinus excelsior*	877.24 Mb	2*n* = 22	867 Mb	89,514	104 Kb	38,852	Illumina HiSeq 2000, MiSeq, Roche 454	Disease resistance	UK	^42^
31	5-May-17	*Rhodiola crenulata*	420.2 Mb	–	344.5 Mb	150,003	144.75 Kb	31,517	Illumina HiSeq 2000/4000	Stress resistance, biosynthesis pathways of medicinal ingredients	China	^43^
32	22-May-17	*Helianthus annuus*	3.6 Gb	2*n* = 34	2.94 Gb	12,318	524 Kb	52,232	PacBio RS II	Flowering time, oil production	France, Canada	^20^
33	24-Jul-17	*Camptotheca acuminata*	516 Mb	2*n* = 44	403.2 Mb	1,394	1.75 Mb	31,825	Illumina HiSeq 2000	Camptothecin biosynthesis	USA	^44^
34	26-Aug-17	*Rhododendron delavayi*	697.94 Mb	2*n* = 26	695.09 Mb	313	637.83 Kb	32,938	Illumina HiSeq 2000	Biosynthesis pathways of medicinal ingredients	China	^45^
35	13-Sep-17	*Apostasia shenzhenica*	471 Mb	2*n* = 68	349 Mb	32	3.029 Mb	21,841	Illumina HiSeq 2000, PacBio	Flower development, seeds without endosperm, evolution of epiphytism	China, Belgium	^17^
36	19-Sep-17	*Rosa multiflora*	711 Mb	2*n* = 14	740 Mb	83,189	90.8 Kb	67,380	Illumina HiSeq 2000, MiSeq	Flower color, flower scent, floral development	Japan	^12^
37	7-Nov-17	*Carnegiea gigantea*	1.3 Gb	–	980.3 Mb	57,409	61.5 Kb	28,292	Illumina HiSeq 2000, MiSeq	Cactus phylogeny	USA	^46^
38	30-Nov-17	*Handroanthus impetiginosus*	557 Mb	2*n* = 40	503.7 Mb	13,206	81.3 Kb	31,688	Illumina HiSeq 2000	Biosynthetic pathway of specialized quinoids	Brazil	^47^
39	1-Dec-17	*Kalanchoe fedtschenkoi*	260 Mb	2n = 34	256 Mb	1,324	2.45 Mb	30,964	MiSeq	Crassulacean acid metabolism	USA	^48^
40	29-Dec-17	*Eschscholzia californica*	502 Mb	2*n* = 12	489 Mb	53,253	752.97 Kb	41,612	Illumina HiSeq 2500	Benzylisoquinoline alkaloid biosynthesis	Japan	^49^
41	25-Mar-18	*Nelumbo nucifera*	–	2*n* = 16	847.16 Mb	14,630	1.48 Mb	30,378	BioNano	Chromosome fusions	China	^50^
42	7-Apr-18	*Phalaenopsis aphrodite*	1.2 Gb	2*n* = 38	1.03 Gb	13,732	0.95 Mb	28,902	Illumina HiSeq 2000/2500	Flower development	China	^15^
43	30-Apr-18	*Rosa chinensis*	560 Mb	2*n* = 14	515 Mb	82	24 Mb	36,377	PacBio RS II, Hi-C	Recurrent blooming, flower scent, and flower color	France	^4^
44	10-May-18	*Bombax ceiba*	809 Mb	–	895 Mb	125	2.06 Mb	52,705	Illumina HiSeq 2000, PacBio RS II	Evolutionary history	China	^51^
45	11-Jun-18	*Rosa chinensis*	532.7 Mb	2*n* = 14	512 Mb	564	3.4 Mb	39,669	Illumina HiSeq 2500, PacBio RS-II	Rickle density, flower petals	France	^5^
46	19-Jun-18	*Salvia splendens*	711 Mb	2*n* = 44	808 Mb	73	3.12 Mb	54,008	PacBio RS II	Flower color, bioactive secondary metabolites	China	^52^
47	13-Jul-18	*Casuarina glauca*	314 Mb	2*n* = 18	283 MB	84	912.67 Kb	26,282	Illumina HiSeq 2000/4000	Nitrogen-fixing root nodule symbiosis	Germany	^53^
48	13-Jul-18	*Cercis canadensis*	301 Mb	–	330 Mb	193	421.03 Kb	34,023	Illumina HiSeq 2000/4000	Nitrogen-fixing root nodule symbiosis	Germany	^53^
49	13-Jul-18	*Mimosa pudica*	896 Mb	–	557 Mb	1,302	119.68 Kb	33,108	Illumina HiSeq 2000/4000	Nitrogen-fixing root nodule symbiosis	Germany	^53^
50	13-Jul-18	*Begonia fuchsioides*	935 Mb	–	374 Mb	569	154.27 Kb	51,638	Illumina HiSeq 2000/4000	Nitrogen-fixing root nodule symbiosis	Germany	^53^
51	4-Sep-18	*Prunus yedoensis*	257 Mb	2*n* = 16	323.8 Mb	519	199 Kb	41,294	HiSeq X Ten, PacBio RS II	S-locus genes	Korea	^10^
52	29-Sep-18	*Lavandula angustifolia*	870 Mb	2n = 50	688 Mb	84,291	96.7 Kb	62,141	Illumina HiSeq 2000	Pathways of isoprenoid metabolism	Canada	^54^
53	17-Oct-18	*Chrysanthemum nankingense*	3.07 Gb	2*n* = 18	2.53 Gb	24,051	130.7 Kb	56,870	HiSeq2000, PacBio RS II	Flower trait, flavonoid biosynthesis	China	^18^
54	14-Nov-18	*Casuarina equisetifolia*	300 Mb	–	301 Mb	366	1.06 Mb	29,827	Illumina HiSeq 2000, PacBio RS II	Secondary growth and DNA modification	China	^55^
55	20-Nov-18	*Osmanthus fragrans*	733.5 Mb	2*n* = 46	740.6 Mb	145	1.59 Mb	45,542	HiSeq X ten, Hi-C	Flower scent	China	^56^
56	17-Dec-18	*Liriodendron chinense*	1.8 Gb	2*n* = 38	1.74 Gb	3,711	3.53 Mb	35,269	Illumina HiSeq 2000, PacBio RS II, Bionano	Systematic evolution of angiosperms	China	^57^
57	18-Dec-18	*Primula vulgaris*	474 Mb	2*n* = 22	411.1 Mb	67,491	294.8 Kb	24,599	Illumina HiSeq 2500	Flower development	UK	^58^
58	2-Jan-19	*Chrysanthemum seticuspe*	3.06 Gb	2*n* = 18	2.72 Gb	354,212	44.7 Kb	71,057	Illumina HiSeq 2000, MiSeq	Flowering time	Japan	^19^
59	28-Jan-19	*Antirrhinum majus*	520 Mb	2n = 16	510 Mb	62	2.62 Mb	37,714	Illumina HiSeq 2000, PacBio RS II	Flower asymmetry, self-incompatibility	China	^59^
60	14-Jun-19	*Sedum album*	305 Mb	2*n* = 48	302 Mb	6,038	93 Kb	44,487	PacBio RS II	Crassulacean acid metabolism	USA	^60^
61	23-Jul-19	*Cerasus yedoensis*	–	2*n* = 16	350.1 Mb	2,292	1.15 Mb	48,280	Illumina HiSeq 2000, MiSeq, HiSeq X	Dormancy- and flowering-associated genes	Japan	^11^
62	23-Jul-19	*Cerasus yedoensis*	–	2n = 16	339.97 Mb	2,279	800 Kb	46,796	Illumina HiSeq 2000, MiSeq, HiSeq X	Dormancy- and flowering-associated genes	Japan	^11^
63	18-Nov-19	*Rhododendron williamsianum*	–	2*n* = 26	532 Mb	11,985	218.8 Kb	23,559	Illumina HiSeq 2000, Hic	Evolutionary history	USA	^61^
64	3-Dec-19	*Tanacetum cinerariifolium*	7.1 Gb	2*n* = 18	7.08 Gb	2,016,451	13.8 Kb	60,080	HiSeq X, HiSeq 4000	Pyrethrin	Japan	^62^
65	6-Dec-19	*Paeonia suffruticosa*	13.66–15.76 Gb	2n = 10	13.79 Gb	499,810	49.94 Kb	35,687	BGISEQ-500, PacBio RS II	MADS-box genes	China	^63^
66	18-Dec-19	*Nymphaea colorata*	433 Mb	2*n* = 28	409 Mb	1,429	2.1 Mb	31,580	PacBio RS II, Hi-C	Flowering transition, flower development, floral scents, flower colors	China	^64^
67	1-Apr-20	*Asparagus setaceus*	720 Mb	2*n* = 20	710.15 Mb	1,393	2.19 Mb	28,410	HiSeq X ten, Hi-C	Resistance R genes	China	^65^
68	14-May-20	*Dionaea muscipula*	3.19 Gb	–	1.5 Gb	104,847	35 Kb	21,135	PacBio RS II	Carnivory genes	Japan, Germany	^66^
69	10-Jun-20	*Chimonanthus salicifolius*	835.5 Mb	2n = 22	820.1 Mb	1,531	2.3 Mb	36,651	Illumina HiSeq 2000, PacBio RS II, Hi-C	Flower development, flavonoid biosynthesis	China	^67^
70	18-Jun-20	*Gardenia jasminoides*	547.5 Mb	2*n* = 22	535 Mb	58,859	44 Mb	35,967	Illumina HiSeq 2000, Oxford Nanopore, Hi-C	Crocin and caffeine biosynthesis genes	China	^8^
71	1-Aug-20	*Forsythia suspensa*	701.40 Mb	2*n* = 28	737.47 Mb	1,214	7.33 Mb	33,062	Illumina HiSeq 2500, Oxford Nanopore	Candidate genes associated with solar radiation, temperature, and water variables	China	^68^
72	10-Aug-20	*Chimonanthus praecox*	778.71 Mb	2*n* = 22	695.36 Mb	1,623	65.35 Mb	23,591	Illumina HiSeq 2000, PacBio RS II, HiSeq X, Hi-C	Floral transition, floral organ specification, early blooming, strong cold resistance, terpene/benzenoid/phenylpropanoid biosynthesis	China	^9^
73	1-Oct-20	*Cerasus serrulata*	256.65 Mb	2*n* = 16	265.40 Mb	304	31.12 Mb	29,094	Illumina X-ten, Nanopore, Hic-C	MADS-box, MYB, WRKY, and plant disease-resistance genes	China	^69^
74	19-Oct-20	*Rhododendron simsii*	525 Mb	2*n* = 26	528.6 Mb	552	36.35 Mb	34,170	PacBio RS II, Hi-C	Metabolic pathways for anthocyanins and carotenoids	China	^70^

### Rosaceae

Rosaceae contains more than 3300 species in 124 genera that are rich in economic and ornamental value and occupy an important position in gardens worldwide. The first flowering ornamental plant to be sequenced was *Prunus mume* (mei) from Rosaceae. In 2009, the National Engineering Research Center for Floriculture of Beijing Forestry University cooperated with the Beijing Genomics Institute (BGI) and other institutions to launch the mei genome project. First, a 237 Mb (84.6% of the estimated genome) genome of wild-type mei was assembled using the Illumina GA II. The scaffold N50 was 577.8 Kb, and 31,390 protein-coding genes were annotated. The genome data were published in *Nature Communications* in 2012, and this effort marked the first genome sequence map of a flowering crop worldwide^3^. Interestingly, equal to the status of mei in China, the “Yoshino cherry” tree (*Prunus × yedoensis*) is one of the most popular *Prunus* species in Japan, and its genome was sequenced by Korean researchers, revealing the parental origin and genomic delimitation of hybrid taxa using both Illumina and PacBio platforms in 2018^10^. Soon afterwards, researchers from Japan also completed two similar genomes of *Cerasus yedoensis*, “Somei-Yoshino”, which were merged into a special genome^11^. At present, a large number of genome studies focusing on *Prunus* and *Rosa* in Rosaceae are underway.

Roses have high cultural and economic value as the most commonly cultivated ornamental and spice plants worldwide. The first ornamental *Rosa* to have its genome sequenced was *Rosa multiflora*, which was reported by Japanese scholars focusing on flower color, flower scent, and floral development traits^12^. Then, another well-known and long-awaited major study was published in *Nature Genetics* in May 2018. A team at the University of Lyon and Centre National de la Recherche Scientifique (CNRS) first revealed another parent of the modern rose, *Rosa chinensis*. The size of the *Rosa* genome is 560 Mb with a contig N50 of 24 Mb, which is one of the most comprehensive plant genomes^4^. Coincidentally, one month later, the same experimental material (a doubled haploid line from ‘Old Blush’) of *Rosa chinensis* was sequenced and republished in *Nature Plants* in June 2018. The high-quality genome was cross-verified, and ornamental and production traits of rose have been interpreted with the joint efforts of many research institutions from France, Belgium, Russia, etc.^5^.

### Orchidaceae

As one of the most abundant families in the plant kingdom, Orchidaceae (orchid) plants are the flagship species of plant diversity protection, known as the “panda of the plant kingdom”. Orchids are divided into five subfamilies: Apostasioideae, Vanilloideae, Cypripedioideae, Orchidoideae, and Epidendroideae. *Phalaenopsis* and *Dendrobium* belong to Orchidoideae and Epidendroideae. *Phalaenopsis* plants are representative of Orchidaceae plants and have important ornamental value. Professor Zhongjian Liu of the National Orchid Conservation Center of China overcame technical problems resulting from high heterozygosity and completed the assembly of the whole-genome sequence of *P. equestris* with a scaffold N50 size of 359.1 Kb. As the first monocot flower for which genome-wide sequencing was completed, the genome of *P. equestris* was published as a cover paper in the journal *Nature Genetics* in November 2014^13^. *Phalaenopsis* is an important potted flower with high economic value worldwide. A 3.1 Gb draft genome assembly of an important winter-flowering *Phalaenopsis* cultivar ‘KHM190’ was completed by researchers from China and Australia^14^. Another species of *Phalaenopsis*, *P. aphrodite*, also underwent high-quality genome sequencing with a scaffold N50 size of 19.7 Mb in April 2018^15^. Scholars from China further analyzed the whole genomes of *Dendrobium officinale* and *Dendrobium catenatuma*, which were published in the journals *Molecular Plant* and *Scientific Reports*, respectively^7,16^. *Apostasia shenzhenica* is representative of one of two genera that form a sister lineage with the rest of the *Orchidaceae*; they have unique flower morphologies as well as diverse lifestyles and habitats. Professor Zhongjian Liu resequenced the high-quality genome of *A. shenzhenica* with a scaffold N50 size of 3.0 Mb. A 349 Mb genome was assembled and published in *Nature* in 2017^17^. *Vanilla fragrans* is a plant of the vanilla family. Due to its unique fragrance that cannot be synthesized artificially, it is known as the “Perfume Queen”. In July 2014, the Fujian Agriculture & Forestry University and National Orchid Conservation Center of China (Shenzhen) officially launched the *Vanilla shenzhenica* genome project. As the first Orchidaceae vine plant to undergo complete sequencing, the genome of *V. shenzhenica* was ~800 Mb with a scaffold N50 size of 288 Kb, and its heterozygosity was ~1.14% (https://www.fafu.edu.cn/2015/0208/c132a18466/page.htm).

### Asteraceae

There are ~24,000–35,000 species in Asteraceae; this family has very high plant diversity, accounting for ~10% of total angiosperms. *Chrysanthemum*, as a typical representative genus, is one of the most important ornamental crops in the world. The genome of *Chrysanthemum morifolium* is estimated to be more than 9 Gb (http://data.kew.org/cvalues/). Since the *Chrysanthemum* genus is large and complex, the genome of *Chrysanthemum* was not reported for a long time. In October 2018, the China Academy of Chinese Medical Sciences, Hubei University of Chinese Medicine cooperated with Nanjing Agricultural University and completed the sequencing of *Chrysanthemum nankingense*, a diploid species (2*n* = 18), which represents one of the progenitor genomes of domesticated chrysanthemums^18^. At around the same time, the de novo whole-genome assembly of *Chrysanthemum seticuspe* was announced by researchers from the Kazusa DNA Research Institute of Japan^19^. The 2.72 Gb of assembled sequences covered 89.0% of the 3.06 Gb *C. seticuspe* genome with 71,057 annotated genes^19^. Sunflower (*Helianthus annuus* L.), in the Asteraceae and the *Helianthus* genus, is a horticultural crop with important economic and ornamental value and a major research focus. In May 2017, a high-quality reference for the sunflower genome was published in the journal *Nature* by scientists from France and Canada^20^. The size of the sunflower genome was 2.94 Gb and covered 80% of the estimated genome; finally, 97% of annotated genes were anchored on a total of 17 pseudochromosomes.

## Resequencing of ornamental plants

Whole-genome resequencing is a process of sequencing the genomes of different individuals of species with known genome sequences and analyzing the differences among individuals or populations. In recent years, to overcome the narrow genetic variation in current ornamental plant breeding programs, genome-scale investigations of wide germplasm panels and cultivated varieties have served to identify important genetic materials to study genomic variation dynamics during domestication and selective breeding^71^. For example, resequencing of multiple materials from different crop species based on genome-wide association study (GWAS) was facilitated to identify key genomic regions associated with plant domestication and selection/improvement^72^. Based on genome-wide resequencing technology, researchers can quickly screen resources, find a large number of genetic variations, and realize genetic evolution analysis and prediction of important candidate genes. Although great progress has been made in the de novo sequencing of ornamental plant genomes, only a few species of ornamental plants, such as sunflower, lotus, mei, rose, sakura, and *Liriodendron chinense*, have undergone genome resequencing (Table 2).

**Table 2:** List of resequenced species of ornamental plants

Code	Date	Species	Plant material	Average sequencing depth	Objects/goals	Reference
1	26-Dec-16	*Fraxinus excelsior*	37 European diversity panel trees	10.9×	Investigating genomic diversity	^42^
2	1-Jun-17	*Helianthus annuus*	80 domesticated lines	10–20×	Evolution of the cultivated sunflower	^20^
3	1-Jun-17	*Helianthus annuus*	72 inbred lines	9.3–19.5×	GWAS	^20^
4	20-Oct-17	*Nelumbo nucifera*	19 individuals	4×	Exploring genomic variation and evolution among different germplasms	^75^
5	27-Apr-18	*Prunus mume*	333 cultivated landraces, 15 wild *P. mume*, and 3 close relatives of *Prunus*	19.3×	Investigating the genetic architecture of floral traits and its domestication history	^74^
6	30-Apr-18	*Rosa chinensis*	8 Rosa species, representing three of the four subgenera (Hulthemia: *R. persica*, Herperhodos: *R. minutifolia* and Rosa).	36.5×	Genetic diversity within the *Rosa* genus	^5^
7	11-Jun-18	*Rosa chinensis*	14 Rosa species, representing three sections (Synstylae, Chinenses, and Cinnamomeae)	5–60×	Gaining insight into the makeup of the genomic relationship of modern roses	^4^
8	4-Sep-18	*Prunus yedoensis*	9 accessions and 7 candidate parental species	7.5–206.3×	Parental origin and genomic delimitation of hybrid taxa	^10^
9	17-Dec-18	*Liriodendron chinense*	14 *L. chinense* individuals and six *L. tulipifera* individuals	24.68–57.35×	Historical demographic fluctuations and present-day genetic diversity	^57^
10	31-Dec-18	*Helianthus annuus*	287 cultivars, 17 Native American landraces, and 189 wild accessions	1–25×	Genetic diversity and to quantify contributions from wild relatives	^73^

Sunflower is not only an ornamental plant but also one of the four major oil crops in the world. In June 2017, genome sequencing of sunflower was completed, eighty domesticated lines (10–20× coverage) and 72 inbred lines (9.3–19.5× coverage) from 480 F_1_ hybrids were resequenced, and 35 genomic regions associated with flowering time were identified by GWAS^20^. Subsequently, to characterize genetic diversity in sunflower and to quantify contributions from wild relatives, scientists from the University of British Columbia sequenced 493 accessions, including cultivars, landraces, and wild relatives^73^. In all, 61,205 genes have been identified within the gene set of the sunflower pangenome, and a large number of candidate resistance genes and single nucleotide polymorphism (SNP) markers for downy mildew resistance were identified by GWAS, which may be of interest to other researchers and sunflower breeders^73^.

To reveal the evolutionary history of *Prunus mume* and the *Prunus* genus and the genetic mechanism of important ornamental characteristics of *P. mume*, 333 cultivated landraces, 15 wild *P. mume*, and three close relatives of *Prunus* (*P. sibirica*, *P. davidiana*, and *P. salicina*) were selected for genome-wide resequencing by Professor Qixiang Zhang from the National Engineering Research Center for Floriculture of China^74^. A total of 5.34 million high-quality SNPs were identified, and 24 important ornamental traits (such as petal color, stigma color, calyx color, bud color, stamina filament color, wood color, petal number, pistil character, bud aperture, and branching phenotype) of 333 cultivars of *P. mume* were analyzed by GWAS for the first time to confirm the hypothesis that *P. mume* exists due to introgression from *P. sibirica* and *P. salicina*^74^.

Three versions of the lotus genome have been published in five years^21,24,50^. To explore the genomic diversity and microevolution related to the rhizome growth pattern, especially the genomic markers of ecotype differentiation, researchers from the Wuhan Botanical Garden of the Chinese Academy of Sciences resequenced 19 individuals including rhizome lotus, seed lotus, flower lotus, wild lotus, Thai lotus and *Nelumbo lutea*^75^. Candidate genes associated with temperate and tropical lotus divergence always exhibited highly divergent expression patterns, which are valuable for the breeding and cultivation of lotus^75^.

Roses have high cultural and economic value because of their outstanding ornamental characteristics and essential oil composition. To analyze the genetic diversity and genetic regulation mechanism of important ornamental traits in roses, eight *Rosa* species representing three of the four subgenera (*R. persica*, *R. minutifolia* and *Rosa*) were resequenced, and the whole-genome sequence of a double-haploid rose line was completed^5^. At the same time, to gain insight into the makeup of modern roses, Raymond et al.^4^ resequenced representatives of three sections (“Synstylae”, “Chinenses” and “Cinnamomeae”) that participated in the domestication and breeding of the modern hybrid rose after the genome of homozygous *Rosa chinensis* ‘Old Blush’ was sequenced.

Sakura (*Prunus yedoensis*) is a woody ornamental plant with important cultural and economic value. To study the genomic relationship between *P. yedoensis* and its closely related species, nine *P. yedoensis* accessions and seven accessions of candidate parental species, including *P. pendula*, *P. jamasakura* and *P. sargentii*, were resequenced and compared to the assembled genome by researchers from Korea^10^. Resequencing data of six related taxa show that 41% of the genes were assigned to the parent state, suggesting that wild *P. yedoensis* is an F_1_ hybrid originating from a cross between *P. pendula* and *P. jamasakura*^10^.

*Liriodendron chinense* is an important woody ornamental plant known as a “woody tulip” in the UK and USA, as its flower shape is similar to that of the tulip. The high-quality genome of *L. chinense* was published in the journal *Nature Plants* in December 2018 in a project led by Professor Jisen Shi from Nanjing Forestry University^57^. To explore the historical demographic fluctuations and present-day genetic diversity between *L. chinense* and *L. tulipifera*, 14 *L. chinense* individuals and 6 *L. tulipifera* individuals were resequenced. Population analysis showed that *Liriodendron* can be divided into three subgroups: the Eastern China subgroup, Western China subgroup and North American subgroup. The species divergence time confirmed that the genetic diversity of *L. chinense* was much higher than that of *L. tulipifera*^57^.

## Applications of whole-genome sequencing in ornamental plants

### Gene annotation

Gene annotation is the process of attributing biological information to the completed sequence of a species using bioinformatics methods. It identifies gene fragments that do not encode proteins, recognizes elements on genes (gene prediction) and adds biological information to the elements for sequence repeat identification, noncoding RNA prediction, gene structure prediction, and gene function annotation. In this way, genes associated with ornamental horticultural traits such as flowering regulation, flower color, floral fragrance, plant type, dormancy, cold resistance, and disease resistance can be identified. The dormancy-associated MADS-box transcription factor (DAM) family, which is related to dormancy induction and release, is especially critical for ornamental plants^76^. Zhang et al.^3^ identified six *DAM* genes in the tandem array in the *P. mume* genome and confirmed that the distribution pattern was consistent with that from previous studies of the peach genome^77^. In *Rosa*, Raymond et al.^4^ identified new candidate genes potentially involved in recurrent blooming, such as *TFL1*, *SPT*, and *DOG1*.

### Comparative genomics research

Based on genome mapping and sequencing technologies, comparative genomics research compares known genes and genome structures to understand the functions of associated genes, their expression mechanism, and the phylogenetic relationships of species. The acquisition of genomic information from multiple closely related species facilitates more comprehensive and in-depth research in comparative genomics. Moreover, it is crucial to perform in-depth comparative analysis of the collinear relationship between the genome sequences of two plants to analyze the origin and evolutionary relationship of plants and to explore important chromosome fragments or gene clusters that control major plant traits, which can provide essential reference information for the discovery and cloning of important genes. Zhang et al. constructed nine ancestral chromosomes of the Rosaceae family by comparing Rosaceae genomes. For the first time, these researchers revealed that ancestral chromosomes have evolved into eight existing chromosomes in *P. mume* via 11 fusions, seven existing chromosomes in strawberry (*Fragaria ananassa*) via 15 fusions and 17 existing chromosomes in apple (*Malus domestica*) via one whole-genome duplication event plus five fusions. These findings lay an important foundation for research to unravel the origin and evolution of Rosaceae^3^.

### Resequencing

Whole-genome resequencing involves the sequencing of genomes in different individuals of species with known genome sequences and subsequent analysis of differences among individuals or populations. Whole-genome resequencing technology can be used to rapidly conduct resource screening, to find a large number of genetic variations and to implement genetic evolution analysis and candidate gene prediction for important traits. These results provide essential references for identifying valuable genetic resources and for horticultural crop breeding and are thus of significant research and industrial value. In *P. mume*, researchers investigated the genetic architecture of floral traits and plant domestication history by resequencing 348 *P. mume* accessions and three other *Prunus* species at an average sequencing depth of 19.3×. Highly admixed population structure and introgression from *Prunus* species were identified in mei accessions^74^. Huang et al.^75^ resequenced and analyzed the genomes of 19 lotus germplasms, provided a reliable and detailed understanding of the genome evolution of different lotus germplasms, and provided clues to key mutations responsible for rhizome enlargement.

### GWAS

A GWAS is a genome-wide comparative analysis or correlation analysis using millions of SNPs in the genome as molecular genetic markers. It is a new strategy to find genetic variations that affect complex traits by comparison. With the development of genomics research and DNA microarray technology, a GWAS can provide an outlined overview of important traits simultaneously and is therefore suitable for the study of complex traits. At the genome-wide level, association studies between genes and traits are conducted with multiple centers, large samples, and repeated verifications. This method has been applied for the screening and identification of major genes for important economic traits in agriculture. In *P. mume*, through a GWAS, researchers have identified significant quantitative trait loci (QTLs) and genomic regions where several genes associated with petal color, stigma color, calyx color, bud color, stamina filament color, wood color, petal number, pistil character, bud aperture, and branching phenotype are located^74^. Taken together, the identification of genetic loci associated with floral and other traits provides more insight into the genetic mechanisms that underlie the domestication of *P. mume* and provides opportunities to design strategies for genomic selection to improve the performance of ornamental species. In sunflowers and roses, the key ornamental trait of flowering time was also identified by the GWAS method^4,20^.

### Comparative analysis with transcriptome data

RNA sequencing is a newly emerging technology that uses next-generation sequencing for transcriptome analysis. It can comprehensively and rapidly acquire sequence information and expression information for almost all transcripts from specific cells or tissues in a particular state, including protein-coding mRNAs and various noncoding RNAs, as well as the expression abundance of different transcripts generated by alternative gene splicing. The transcriptome is an inevitable link that connects genetic information of the genome with the biological functions of the proteome. Currently, transcriptional regulation is the most well-studied and foremost regulatory method in organisms. Transcriptome studies are the foundation and starting point of gene function-structure studies and the first issue to address after the completion of whole-genome sequencing. Furthermore, transcriptome analysis provides large numbers of molecular markers, such as simple sequence repeats and SNPs. All of the sequence information, expression data, and molecular markers facilitate the localization of QTLs for key ornamental traits in ornamental plants through genetic mapping and contribute to the development of molecular markers in close linkage with excellent traits for use in the molecular marker-assisted breeding of flowers. Based on the genome sequence of *P. mume*, vital differences in gene expression between the bud stage and squaring stage were observed, and 7,813 DEGs were identified, which provided a special perspective on floral scent formation in *P. mume*^78^. The water lily genome revealed variable genomic signatures of ancient vascular cambium losses, and the expression profiles of floral ABCE genes, floral scent and color genes were screened from the DEGs in a comparative analysis of the transcriptome^64^.

### Development of SNP microarrays

According to their position in genes, SNPs can occur in coding regions, noncoding regions, and gene spacer regions. They are DNA molecular markers that have the most abundant polymorphisms in the genome and are characterized by large numbers, a uniform distribution, and easy typing. SNPs can be used for the identification of genetic variation and genotyping of associated phenotypes. Using SNPs as molecular markers to construct genetic variation maps of the genome has become a vital part of the research for studying genome diversity, obtaining domesticated selection regions, and screening key genes of important traits. Based on the genome sequence and resequencing of *P. mume*, a total of 1,298,196 raw SNPs were located within coding regions of genes, 733,292 of which were nonsynonymous^74^. Furthermore, by combining transcriptome data, 76 SNPs within DEGs were identified that were associated with petal, stigma, calyx, and bud color^74^. In *sacred lotus*, wild and Thai lotus exhibited greater differentiation with a higher genomic diversity than cultivated lotus based on SNP sites in resequenced species^75^.

## Exploiting genes associated with important ornamental traits

During the course of whole-genome sequencing, a very large number of genes, in the range of 19,507–87,603, are annotated for each flowering species (Table 1). Through further analysis, important genes associated with floral development, flower color formation, and stress resistance can be discovered. This is conducive to the breeding of unique, high-quality, and high-resistance varieties or types of a species and provides important references for improving ornamental and resistance qualities in other flowering species.

### Candidate genes for controlling floral development

Flower blooming is a process that involves the formation of inflorescence meristems and flower meristem tissues through floral induction and a series of internal and external factors, followed by the generation of floral organ primordia and eventually the release of flora bud dormancy to form floral organs. The process of flowering is controlled by a complex regulatory network, with at least seven flowering regulation pathways found in *A. thaliana*^79^. The genes associated with floral development can be divided into two classes. One class consists of genes that control the formation of inflorescence meristems and determine the direction of newly formed floral primordia. These genes influence the flowering time of plants by controlling the formation of inflorescence meristems or flower meristems, and mutations in these genes can result in earlier or later flowering mutants. The other class consists of genes that determine the formation of floral organs, and mutations in these genes can result in homeoboxes^79^. In ornamental plants, the morphology and number of floral organs have undergone substantial variations, for example, double petals, multiple sepals, and multiple pistils and stamens, developing into independent flowers during the course of long-term artificial domestication and cultivation. These variations increase the ornamental value of ornamental plants while providing excellent materials for the study of floral organ development in plants. With genomic data analysis, as an important scientific issue, some key genes related to flowering transition and flower development have been analyzed, such as those in *Tarenaya hassleriana*^23^, *Dendrobium officinale*^7^, *Primula veris*^28^, *Dendrobium catenatum*^16^, *Hibiscus syriacus*^41^, *Rosa*^4,5,12^, *Chrysanthemum*^18,19^, and *Nymphaea colorata*^64^.

### Candidate genes for controlling anthocyanin synthesis

Flower color is one of the most vital quality traits of ornamental plants. Anthocyanin is an essential pigment for coloring flowers, and its biosynthesis is catalyzed by a series of enzymes^80^. Various anthocyanins are formed due to differences in the substituent groups at varied positions on the basic skeleton, thus leading to different plant organ colors, such as red, purple, blue-purple, and blue. Anthocyanins are flavonoid secondary metabolites in plants and the most widely distributed water-soluble pigments in nature, playing a major role in the color formation and antioxidation in plant flowers and fruits. R2R3-MYB genes are involved in anthocyanin synthesis^81^. In *P. mume*, 96 R2R3-MYB genes were identified and divided into 35 subfamilies. Finally, the functions of *PmMYB1* and *PmMYBa1* were identified by overexpression in tobacco and significantly promoted the accumulation of anthocyanins in transgenic tobacco. The flower colors of *PmMYB1*-overexpressing transgenic plants were significantly deepened, and the anthocyanin contents in the corolla of transgenic plants were significantly higher than those of the control^82^. To understand the molecular basis of the blue color in water lily, delphinidin 3′-O was identified as the main blue anthocyanidin pigment, and some genes for an anthocyanidin synthase and a delphinidin-modification enzyme were screened by comparing the expression profiles between two *N. colorata* cultivars with white and blue petals^64^. Interestingly, after the butterfly pea UDP (uridine diphosphate)-glucose: anthocyanin 3′,5′-O-glucosyltransferase gene was introduced in chrysanthemums, blue flowers appeared^83^. In *Rosa rugosa*, two MYB transcription factors have been confirmed to affect flower color by regulating flavonoid biosynthesis in response to wounding and oxidation^84^. In *Paeonia*, a *chalcone synthase* (*PhCHS*) involved in flavonoid biosynthesis and two *anthocyanin O-methyltransferase* (*AOMT*) genes were consistent with anthocyanin accumulation in petals^85,86^.

### Candidate genes for controlling floral scent biosynthesis

Floral scent, as one of the quality traits of ornamental plants, has great aesthetic, economic, and application value. The scent components present in petals primarily include secondary metabolites such as esters, alcohols, ketones, aldehydes, terpenes, and volatile phenols, mainly derived from terpene metabolism, phenylpropane metabolism, and the lipoxygenase pathway^87^. There are various types of scent components in different petals, thereby forming distinct scents among various flower species. In a study on the molecular mechanism responsible for the floral scent in *P. mume*, Zhang et al.^3^ first discovered that the *benzylalcohol acetyltransferase* (*BEAT*) gene can directly catalyze the formation of benzyl acetate, a crucial component of the floral scent in *P. mume*. Moreover, based on genomic data from *P. mume* and *P. persica*, 44 unique *PmBEATs* were found in *P. mume*, far more than the 16 in apple, 14 in strawberry, and four in grape. These *PmBEAT* genes originated from gene duplication events during the species evolution of *P. mume*, and retroduplication and tandem duplication were the two dominant duplication patterns. Overexpression of the *PmBEAT36* or *PmBEAT37* genes increased benzyl acetate production in the petal protoplasts of *P. mume*, and interference in the expression of these genes slightly decreased the benzyl acetate content^88^. Zhao et al.^78^ conducted a comparative transcriptome analysis of different developmental stages and tissues of flower genes associated with floral traits and preliminarily selected 12 new genes involved in floral scent formation in *P. mume*. Furthermore, five of the TFs (*bHLH4*, *bHLH6*, *bZIP4*, *ERF1*, and *NAC1*) from *Phalaenopsis bellina* have been proven to be involved in orchid floral monoterpenes^89^. In *Plumeria rubra*, *PrCYP79D73* is involved in floral volatile organic compounds and other nitrogen-containing volatiles^90^.

### Candidate genes for controlling plant architecture

Rich and diverse plant architectures are the result of long-term evolution, natural selection, and a complex regulatory process of interaction between genetics and the environment. Diverse plant architecture traits are not only conducive to the creation of rich and diverse horticultural landscapes but are also favorable for plant adaptation to complex environments and competition and the utilization of light and nutrients. Along with the completion of whole-genome sequencing for multiple ornamental plants of the genus *Prunus*, the results lay an important data foundation for studying the molecular genetic mechanisms of pendulous traits^3,91^. According to the eight scaffolds of the *P. mume* genome, Zhang et al. constructed a high-density genetic map using specific-length amplified fragment sequencing (SLAF) and mapped QTLs for major traits such as plant type, flower color, petals, and leaves in *P. mume*. They found 10 SLAF markers that were closely linked to the pendulous traits of *P. mume*. Using these markers, the pendulous traits were finely mapped to a 1.14 cM region on chromosome 7, and 36 candidate genes that might be associated with the pendulous traits of *P. mume* were predicted^92^. Breakthroughs were also achieved in the mining and labeling of genes for weeping and dwarf traits in peach (*P. persica*) by using genome and bulked segregant analyses^93^.

### Candidate genes for controlling dormancy release

Flowers of the genus *Prunus*, such as *P. mume* and *P. yedoensis*, are early flowering types in spring. Zhang et al.^3^ explored the molecular mechanisms underpinning dormancy break and flowering in *P. mume* at low temperature. These researchers identified a total of six *dormancy-associated MADS-box* (*DAM*) genes with a tandem repeat distribution in the genome. The six *DAM* genes in *P. mume* are derived from a series of duplication events in the following order: *PmDAM1*, *PmDAM3*, *PmDAM2*, *PmDAM5*, *PmDAM4*, and *PmDAM6*. The molecular evolution pattern of *DAM* genes is unique to *Prunus* plants and is present in *P. persica*, but tandem genes have not been found in *M. domestica* or *F. ananassa*. This phenomenon could be related to the earlier flowering of *Prunus* plants, including *P. persica*, *P. mume*, apricot (*Armeniaca vulgaris*) and sweet cherry (*Prunus avium*), than of most other flowering species^3^. *DAM* genes are regulated by C-repeat-binding transcription factors (CBFs). A conserved CBF site was found 1000 bp upstream of the transcription start site of *DAM4*-*DAM6* in *P. persica* and plum (*Prunus salicina*). The latest research results show that a sense-response relationship between PmCBFs and PmDAMs is exhibited in cold-induced dormancy and is jointly regulated by six PmCBFs and PmDAM4–6^94^.

### Candidate genes for controlling self-incompatibility

Self-incompatibility has always been an important research topic in the molecular genetic biology of flowers. According to different hereditary patterns of pollen incompatibility phenotypes, the regeneration disorder whereby plants reject self-pollen can be divided into sporophytic self-incompatibility and gametophytic self-incompatibility^95^. Various flowers of the Rosaceae family, including *P. mume*, *P. yedoensis* and *P. persica*, all exhibit gametophytic self-incompatibility, which is controlled by an S-locus with multiple alleles, including two linked genes: one is the S-RNase gene specifically expressed in pistil tissue, and the other is the S-haplotype-specific F-box gene specifically expressed in pollen^96^. In *Tarenaya hassleriana*, three syntenic regions containing most of the genes of the S-locus were found, and it was assumed that the single-copy ancestral region contained homologs of *Pub8*, *ARK3*, and *B120*^23^.

### Candidate genes for controlling disease resistance

Disease resistance is an essential trait that attracts research attention across all flowering plants. Thus, the whole-genome analysis also focuses on the genes associated with disease resistance. The genes involved in plant disease resistance are mainly R genes, which encode proteins with extremely high structural similarities, such as leucine zippers, nucleotide-binding sites, transmembrane domains, leucine-rich repeats, and similar extracellular regions of drosophilid toll protein and mammalian toll and interleukin-1 receptor (TIR). Nucleotide-binding site leucine-rich repeat genes constitute the gene family with the widest distribution and largest number of plant R genes. In their encoded proteins, the nucleotide-binding site is present near the N-terminus, while the leucine-rich repeat exists near the C-terminus. The N-terminus of proteins encoded by different genes may also include one or more of the following two conserved structures: the coiled-coil motif and TIR motif. In the *P. mume* genome, 253 leucine-rich repeats receptor-like kinase (LRR-RLK) genes were identified, and most pathogenesis-related (PR) gene families were notably expanded and arranged in tandem, especially PR10^3^. In *Hibiscus syriacus*, resistance (R) genes account for 0.53% of its total predicted genes, which is lower than that of other plants evaluated in genomic studies (0.63 to 1.35%)^41^. The *Asparagus setaceus* genome included 76 R genes with nucleotide-binding sites (NBSs), and the R genes belonged to five groups: TIR-NBS, CC-NBS-LRR, NBS-LRR, NBS, and CC-NBS. NBS-LRR was the largest group, including a total of 29 genes^65^.

### Candidate genes for controlling abiotic stress resistance

Adverse conditions such as low temperature, humidity, heat, drought, and saline-alkali conditions severely inhibit the growth and development of ornamental plants. These conditions can cause changes in plant physiology, biochemistry, and morphology and even lead to death. Due to this issue, cultivation facilities for ornamental plants are cumbersome and cannot be widely promoted, which considerably affects their qualities and benefits. Low temperature is an important factor that constrains the normal growth, development, and geographical distribution of plants. Stress caused by low temperature can be divided into chilling stress (>0 °C) and freezing stress (<0 °C). Plants from the tropics and subtropics are more sensitive to cold; in contrast, plants from temperate regions have evolved complex mechanisms to resist and adapt to chilling (freezing) stress, protecting the plants from injury. Cold acclimation is a responsive protection mechanism for plant adaptation and resistance to low-temperature stress, and this process is regulated by a complex network^97^. In particular, the CBF pathway is considered the most important and well-studied pathway^98^. Based on the genome data for *P. mume*, 30 *LEA* genes were identified, and heterologous expression of *PmLEA* increased the cold resistance of *Escherichia coli* and tobacco (*Nicotiana tabacum*)^99,100^. Furthermore, a molecular regulation model of the *PmDAM* and *PmCBF* genes in response to dormancy and dormancy release of flower buds induced by low-temperature signals was proposed based on yeast two-hybrid and bimolecular fluorescence complementation experiments^94^.

## Prospects for whole-genome sequencing data for ornamental plants

The Earth BioGenome Project (EBP) is a massive project in biology that aims to sequence, catalog, and characterize the genomes of all of Earth’s eukaryotic biodiversity over a period of 10 years. For plants, the core scientific problems are to improve crop yields and other agronomically important traits, biofuel production, gene editing, and conservation of endangered species^101^. The 10,000 Plant Genome Sequencing Project (10KP) initiated by the Beijing Genomics Institute in Shenzhen (BGI-Shenzhen) is a landmark effort to catalog plant genomic variation and represents a major step in understanding the tree of life^102^. A tentative plan of the 100 Flowers Genome Sequencing Project has been put forward by the National Engineering Research Center for Floriculture in China. Many ornamentals are marked by high ploidy levels and homologous polyploids (chrysanthemum and alfalfa) or extremely large genome sizes (lily and tulip), which limit the development and utilization of genome sequencing technology in ornamental plants. Along with the development of sequencing and bioinformatics analysis technologies and the continuous emergence of various new biological technologies, genomics research on ornamental plants has developed faster and better. Although genome sequencing and assembly of flowering plants face substantial difficulties, the quality of genome assembly results is relatively high in terms of the analytical results from 69 flower species that underwent genome sequencing, and four of them have been resequenced using updated sequencing technology^5,11,37,50^. As far as we know, there are at least a dozen ornamental plants undergoing the process of genome quality improvement. As more ornamental plant genomes are sequenced, further bioinformatics analysis could reveal crucial basic information on the origin of species and the genes that control flower traits. The development of genomics will surely address the knowledge gaps of traditional breeding methods. The ultimate goal is to obtain the optimal type of flower variety with fixed-point improvement and the aggregation of multiple elite traits by using the most effective and rapid method.

China has 30,000 species of higher (flowering) plants, and some ornamental flowering plants reached Europe quite early^103^. Chinese people love flowers and cultivate many kinds of brilliant flowers, such as mei, peony, chrysanthemum, rose, lily, lotus, and orchid. Due to the rapid development of genome sequencing technology worldwide, large quantities of whole-genome sequencing data are in urgent need of deep mining. A long-term strategic genomics research plan should be formulated that is not limited to cultivated species but considers thorough development of the sequencing of important wild relatives of ornamental species in China and promoting the mining, protection, and utilization of important genetic resources. It is essential to put an end to the dependence on the apparent phenotype, transform investigations into genotype-dependent research and shift from single-gene studies to GWAS. Efforts should be made to vigorously promote the application of genomics in gene cloning and molecular breeding in China and to improve the breeding capacity and level of horticultural crops.

Due to their complexity and particularity, plant genomes have always been an important focus of genomics. Before the second generation of high-throughput sequencing, sequencing costs were high, and the throughput was low. For species with highly repetitive sequences, it was too difficult or too expensive for researchers to obtain the whole-genome sequences of high repeat sequence species. Many species with important economic and ornamental value have not yet been submitted to complete genome sequencing. In short, due to the particularity and diversity of ornamental plants, there are challenges and opportunities in genome research of these species. Challenge: (1) Complex genome. The term complex genome refers to a kind of genome that cannot be directly analyzed by conventional sequencing and assembly methods. It usually refers to a genome containing a high proportion of repetitive sequences, high heterozygosity, extreme GC content, and difficulty in eliminating foreign DNA contamination. (2) Autopolyploidy. Autopolyploidy is common in ornamental plants. It is usually formed by doubling two or more sets of genomes, which is of great value in genetic breeding and agricultural production. Using conventional methods, it is easy to connect incorrect allele fragments together, resulting in the wrong connection of homologous chromosomes and a large number of chimeric assemblies; thus, assembly is still difficult. (3) Megagenome. Megagenome generally refers to species with genomes larger than 10 Gb. The sequencing and analysis of these species are very involved, especially for assembly analysis, which is a major challenge. *Paris japonica* is an unusual plant. Scientists have found that it has the world’s largest genome, with 150 Gb, which is 50 times more than that of humans. Although the genomes of some ornamental plants have been deemed complete, the assembly quality of some species is poor, and a small number of “holes” have not yet been completed due to technical limitations, although the interest of scientists in this regard is debatable. The latest research shows that the sequences that were once considered irrelevant, or “garbage”, in the genome have their own significance. These missing sequences play a very important role, and we now have the opportunity to mine them. Third-generation sequencing technology (PacBio and Nanopore) can make up for the holes in some genomic regions that are difficult to assemble due to sequencing errors, repeat regions, heterochromatin, genomic polymorphisms, and second-generation sequencing preferences. To solve the challenge of sequencing the genomes of ornamental plants, the following new technologies can be tried with third-generation sequencing technology. (1) Pangenome. The pangenome includes the core genome and the nonessential genome. Among them, the core genome refers to the genes that exist in all individuals; the nonessential genome refers to the genes that exist only in some individuals. (2) Hi-C. The advantages of Hi-C sequencing technology are as follows: on the one hand, there is no need to construct a large number of F_1_ populations, as only individuals are needed; on the other hand, the haplotype genome can be separated without parent purification, so this method is suitable for the assembly of a highly heterozygous genome that is not easy to purify.

With the development of sequencing technology, the concepts of difficult genome sequencing and assembly quality have also developed and changed. We cannot sequence everything for the sake of genome sequencing. The purpose of sequencing must be to reveal the key scientific problems of species. We should strengthen research related to transcriptomics, metabolomics, proteomics, degradomics, and phenomics. With more genomic data published, it has become a great challenge to analyze, store and share the massive amounts of genome sequencing data. A key problem is how to solve the time and cost problems faced by researchers to achieve the purpose of reducing repetitive research, improving the practicability of scientific research, mining research content, and improving the transparency of scientific research and data sharing with cross-research into other fields. Moreover, it is necessary to enhance bioinformatics education and apply bioinformatics in practice. With the continuous development of sequencing technology, we believe that the whole-genome sequencing of horticultural crops will enter a rapid development stage in the near future, leading to tremendous contributions to the world’s horticultural industry.
